# A randomised controlled trial on the Four Pillars Approach in managing pregnant women with anaemia in Yogyakarta–Indonesia: a study protocol

**DOI:** 10.1186/1471-2393-14-163

**Published:** 2014-05-07

**Authors:** Widyawati Widyawati, Suze Jans, Hans Bor, Rukmono Siswishanto, Jeroen van Dillen, Antoine LM Lagro-Janssen

**Affiliations:** 1 School of Nursing, Faculty of Medicine, Universitas Gadjah Mada, Yogyakarta, Indonesia; 2 Department of Primary and Community Care, Gender & Women’s Health, Radboud University Medical Centre Nijmegen, Nijmegen, The Netherlands; 3 Royal Dutch Organisation of Midwives (KNOV), Utrecht, The Netherlands; 4 Department of Obstetrics and Gynaecology, Sardjito Hospital-Medical Faculty, Universitas Gadjah Mada, Yogyakarta, Indonesia; 5 Department of Obstetrics and Gynaecology, Radboud University Medical Centre Nijmegen, Nijmegen, The Netherlands

**Keywords:** Four Pillars Approach, Healthy life style, Social support, Nurse-midwives’ competencies, Professional behaviour, Pregnant women, Anaemia, Antenatal care

## Abstract

**Background:**

Anaemia is a common health problem among pregnant women and a contributing factor with a major influence on maternal mortality in Indonesia. The Four Pillars Approach is a new approach to anaemia in pregnancy, combining four strategies to improve antenatal and delivery care. The primary objective of this study is to measure the effectiveness of the Four Pillars Approach. The barriers, the facilitators, and the patients’ as well as the midwives’ satisfaction with the Four Pillars Approach will also be measured.

**Methods/Design:**

This study will use a cluster randomised controlled trial. This intervention study will be conducted in the Public Health Centres with basic emergency obstetric care in Yogyakarta Special Province and in Central Java Province. We will involve all the Public Health Centres (24) with emergency obstetric care in Yogyakarta Special Province. Another 24 Public Health Centres with emergency obstetric care in Central Java Province which have similarities in their demographic, population characteristics, and facilities will also be involved. Each Public Health Centre will be asked to choose two or three nurse-midwives to participate in this study. For the intervention group, the Public Health Centres in Yogyakarta Special Province, training on the Four Pillars Approach will be held prior to the model’s implementation. Consecutively, we will recruit 360 pregnant women with anaemia to take part in part in the study to measure the effectiveness of the intervention. The outcome measurements are the differences in haemoglobin levels between the intervention and control groups in the third trimester of pregnancy, the frequency of antenatal care attendance, and the presence of a nurse-midwife during labour. Qualitative data will be used to investigate the barriers and facilitating factors, as to nurse-midwives’ satisfaction with the implementation of the Four Pillars Approach.

**Discussion:**

If the Four Pillars Approach is effective in improving the outcome for pregnant women with anaemia, this approach could be implemented nationwide and be taken into consideration to improve the outcome for other conditions in pregnancy, after further research.

**Trial registration:**

Current Controlled Trials ISRCTN35822126.

## Background

Anaemia is a significant health problem among pregnant women in Indonesia with a major impact on maternal mortality [1]. The World Health Organization (WHO) defines anaemia as a condition where the level of haemoglobin (Hb) in the blood is less than 11 g/dl [2]. Nutrional anaemia in pregnancy is found to be the most prevalent in Indonesia [3]. Other factors causing anaemia such as HIV, malaria and hookworm infection are also found in several areas in Indonesia [4,5]. One of the endemic areas for malaria is Kulonprogo, one of the districts in Yogyakarta Special Province [5].

The 2007 Indonesia Demographic and Health Survey reported the prevalence of anaemia in pregnancy of 28% [6]. A similar prevalence was found in Yogyakarta in 2009 [7]. Concerning maternal health indicators in Indonesia, only 59.8% of births are attended by skilled birth attendants [8] and less than 70% of pregnant women attend no more than four antenatal care visits [9]. The antenatal care use is also influenced by the knowledge of patients and other family members [10].

Studies on maternal health services and the quality of nursing-midwifery care in Indonesia, found that nurse-midwives have a lack of knowledge and skills to identify the risk factors in pregnant women, and that their professional behaviour is sub-standard [11-13]. Also the antenatal care attendance of pregnant women is low, resulting in a poor quality of antenatal care [14]. The studies suggested that antenatal care training for nurse-midwives be improved, as an important strategy to help solve these problems [11-14]. One of the studies showed that women have negative perceptions about the quality of maternity care, caused by the nurse-midwives’ impolite, negligent behaviour and intentional humiliation of the women (such as verbal abuse) [13].

Studies from other low-income countries suggested the importance of counselling and health education for pregnant women with anaemia to improve their knowledge and awareness about a healthy pregnancy [15-19]. One study advised that booklets should be given to increase women’s knowledge about Iron Deficiency Anaemia (IDA) and mother and child health [16]. All studies expressed an urgent need for training programs for nurse-midwives to improve the quality of health services, including the detection of anaemia risk during pregnancy [16,17].

The WHO stressed the importance of antenatal care visits to maintain the health status of the mother and the wellness of the foetus [20]. A study which evaluated the effectiveness of an early antenatal health promotion workshop found that a healthy lifestyle during pregnancy correlated with maternal and infant health outcomes [21]. The support of the husband or other family members, and a caring attitude from the nurse-midwife proved to motivate pregnant women to attend the available antenatal care services [22-24].

Based on the result of these studies, experts’ opinion and indepth interviews with nurse-midwives, we designed a Four Pillars Approach to synergize the empowerment between pregnant women and nurse-midwives. These four pillars are: a healthy lifestyle during pregnancy, social support from the husband or other family members, adequate knowledge and skills of the nurse-midwives, and the nurse-midwives’ professional behaviour.

The first and second pillars (healthy lifestyle and the strengthening of social support) represent patient empowerment. The nurse-midwives empowerment is represented by the third and fourth pillars: adequate knowledge and skills, and the professional behaviour of nurse-midwives.

In this study, we will evaluate the effect of the Four Pillars Approach on pregnant women with anaemia.

### Objectives

#### Primary objective

To measure the effectiveness of the Four Pillars Approach in the management of pregnant women with anaemia.

#### Secondary objective

To investigate the barriers and facilitating factors of the implementation of the Four Pillars Approach, as well as nurse-midwives’ satisfaction with the approach.

## Methods/Design

### Study design

This study will use a cluster randomised controlled trial design [25], measuring outcomes on individual level of the included pregnant women with anaemia. Individual outcomes between intervention and control group will be compared.

The nurse-midwives involved in the intervention group will be trained in the Four Pillars Approach prior to the implementation of this model. The nurse-midwives will follow a refresher course on current management of anaemia in pregnancy, therapeutic communicaton (counselling) and professional behaviour. They will also have practical guidance in the skill laboratory phase where they have to demonstrate their knowledge and skills to manage pregnant women with anaemia such as taking laboratory tests, carry out physical examination on the signs and symptoms of anaemia, and communcation in terms of giving health education to patients.

Then, the trained nurse-midwives in all Public Health Centres with emergency obstetric care in the intervention group will provide antenatal care services based on the Four Pillars Approach to the pregnant women with anaemia. Meanwhile, in the control group all Public Health Centres with emergency obstetric care will provide their usual antenatal care services to the pregnant women with anaemia.

In an intervention study, the effectiveness of antenatal care given by trained nurse-midwives to pregnant women with anaemia, following the Four Pillars Approach, will be compared to the usual care given. The usual care is the routine antenatal care carried out by nurse-midwives with a three year diploma in nursing-midwifery education.

### Setting

The Yogyakarta Special Province has a total of 24 Public Health Centres with emergency obstetric care which we will use in our study. Based on the population criterias (such as the prevalence of anaemia in pregnancy, cultural background, health insurance), demographic characteristics (such as accessibility and location of the Public Health Centre), and facilities (such as laboratory, medical devices, and emergency kit) available in the Public Health Centre with emergency obstetric care, we will choose another 24 Centres (the same number as are in Yogyakarta) for our control group. Central Java Province has many similarities with Yogyakarta Special Province. The Provincial Health Offices of Central Java Province gave us the information about the Public Health Centres with obstetric emergency care in some districts of Central Java Province which surround Yogyakarta Province, and which we could use as research fields. Based on this information, we will randomly choose the 24 Public Health Centres for our control group. In total, we will involve 48 Public Health Centres as our research fields. Generally, there are about seven to ten nurse-midwives in every Public Health Centre eligible, according to the inclusion criteria. Considering the other health services that should be handled by the nurse-midwives in Public Health Centre and the activities that should be conducted by the nurse-midwives if they are involved in the study, the head of Public Health Centres permitted to involved only two or three nurse-midwives in this study. These are the nurse-midwives who will be trained and who will treat all pregnant women with anaemia according to the study protocol. The nurse-midwives who have been selected, still have the right not to participate in our study. We estimate 90% of nurse-midwives will join our study.

### Participants

#### Nurse-midwives

The inclusion criterias for the nurse-midwives in the intervention and control groups are: they hold a three year diploma in nursing-midwifery education, and they work on a daily basis in Public Health Centres with basic emergency obstetric care. All participants will be required to sign a consent form.

#### Study population

The women who attend antenatal care in the Public Health Centres will be included consecutively. Their inclusion criteria are: pregnant women with a Hb of less than 11 g/dl in the first trimester of pregnancy and who are living with their husband or other family members. The pregnant women with severe anaemia (Hb less than 7 g/dl), would first be referred to a doctor, then she could be included, if she does not need to be hospitalized. Pregnant women over twelve weeks of gestation will be excluded. Regarding to the agreement of patient management in our country, the pregnant women who infected by HIV, malaria, or hookworm should get the medical treatment from the specialist doctor. Therefore, women who are infected by HIV, malaria or hookworm will also be excluded.

Based upon the number of participants needed, the nurse midwives will be asked to recruit eligible pregnant women with anaemia. All participants will be required to sign a consent form to agree to participate in the study.

### Intervention

#### Training of the Four Pillars Approach

This training will be given to the nurse-midwives in order to introduce the new concept of the Four Pillars Approach in managing pregnant women with anaemia, and training them how to implement this model in Public Health Centres with emergency obstetric care. Based on international literature and experts’ opinion, we will develop a training module for the Four Pillars Approach in managing pregnant women with anaemia. At the end of this training, the nurse-midwives will have the knowledge and skill to manage pregnant women with anaemia based on the Four Pillars Approach.

The training will last eight hours, and consist of two sessions. The first session will be held in-class, and the second session will take place in the skills laboratory of the Nursing School of the Universitas Gadjah Mada Yogyakarta. A specialist in obstetrics and gynaecology, a senior teacher and a senior nurse will take part as teachers during the in-class session. Different educational methods will be used, such as power point slides, video and role play. In the skills laboratory session, training participants will be divided into six groups; each group consisting of ten to eleven participants. Every group will be directed by one tutor and one “simulated patient” (a person who acts like a pregnant women with anaemia). Case scenario, drama, demonstration and role play will be used to capture a real-life situation. All participants will be assessed and are required to pass this. The tutor will observe the competency of each nurse-midwife, and give an assessment to each participant based on the observation check list. There are four items to be observed by the tutor: procedure of treatment, communication, professional behaviour, and data registration. Those items are scored by using a Likert scale 1 – 3. Score 1 will be given if the performance of the nurse-midwife is unsatisfactory, score 2 if the performance is reasonable but could still be improved upon, and score 3 if the performance of the nurse-midwife is excellent and complete according to the protocol. All the nurse-midwives are required to pass this assessment exam with a minimum score of 60%.

#### Training module and booklet

A training module will be given to every nurse-midwife involved in the training programme. The training module consists of general information about the training, training materials (such as: physiological changes during pregnancy, anaemia in pregnancy, laboratory testing, the concept of the Four Pillars Approach, and professional behaviour for the nurse-midwife), the Four Pillars Approach protocol, and samples of reporting and Case Report Forms (CRF).

The booklets will be given to all pregnant women with anaemia in the intervention group, in order to increase their knowledge on the prevention of anaemia in pregnancy. The booklet will be designed with text and pictures to improve understanding of the information. It will also include a check list table to monitor and record the intake of iron tablet supplements, folic acid and vitamins. The husband or family member will be asked to remind the pregnant woman to take the tablets and record this in the check list.

#### Parenting class

In the intervention group, the husband or other family member will be asked to attend the antenatal care visits together with the pregnant woman and to accompany the pregnant woman to two parenting classes. During the first parenting class, the trained nurse-midwives will explain the content of the booklet. In the second parenting class, the trained nurse-midwives will offer the opportunity to the pregnant women and her companion to share their experiences. The duration of each parenting class will be one hour.

### Data collection

#### Characteristics of pregnant women

The nurse-midwives will collect the data of patient’s individual characteristics (such as: age, parity, job, the distance to the Public Health Centre from the patient’s house, and the availability of health insurance), medical history (such as: obstetric history, family health history), data about the current pregnancy (such as: Hb, antenatal examination results, parenting class attendance); and documentation relating to the number of antenatal visits, the number of non participants and reasons why, and the number of drop outs and reasons why.

#### Outcome measures

##### *Primary outcome*

Hb level, and the number of antenatal care attendances and skilled birth attendance at delivery are the primary outcomes of this study.

The Hb level will be measured before the twelfth week of gestation (T0) and between week 35 and 37 of gestation in the third trimester (T1). The difference between T0 and T1 in the intervention group will be compared with the difference in the control group. The antenatal care attendance will be counted based on the documentation of the nurse-midwives at the end of pregnancy. Skilled birth attendance at delivery will be identified from the patient’s medical records in the Public Health Centre or hospital. All primary outcomes of the intervention group will be compared to those in the control group.

##### *Secondary outcome*

The barriers and facilitating factors of the implementation of the Four Pillars Approach, as well as nurse-midwives’ satisfaction with the approach are the secondary outcomes.

Focused group discussion will be conduct to investigate the barriers and facilitating factors. The trained nurse-midwives, and the nurse-midwives coordinators will be involved in the focused group discussion. The nurse-midwives’ satisfaction about the implementation of the Four Pillars Approach will be measured concerning the quality of health services, procedural clarity, and communication between the nurse-midwives and patients, by using the questionnaire of Langer A [26] with some modification to adapt it to the local situation.

### Analysis plan

#### Intervention model development

Studying relevant literature, consulting experts in this field and indepth interviews with the nurse-midwives will be conducted to evaluate the implementation of the current antenatal care standard and to investigate nurse-midwives experiences in managing pregnant women with anaemia in Public Health Centres. The result of those activities will be used as a knowledge base for designing the new model for managing pregnant women with anaemia in Public Health Centres.

#### Monitoring of the study

Firstly, we will invite nurse-midwives, the heads of Public Health Centres, and the coordinators of the family health programme from the Health District Offices in Yogyakarta Special Province, to attend a one day seminar. The aim of this seminar is to disseminate information, and to give the feed back, on the concept of the new model for the Four Pillars Approach in managing pregnant women with anaemia. Secondly, the revisions of the new model will be done when it is needed, based on the result of the seminar discussions. Thirdly, we will develop a team trainer for the Four Pillars Approach in managing pregnant women with anaemia, which will consist of one obstetric and gynaecologist, two maternity nurse specialists, and six senior nurses. Then, the team trainer will give a one day training course on the Four Pillars Approach in managing pregnant women with anaemia to the nurse-midwives. At the end of the training, the trainers will evaluate the nurse-midwives’ knowledge and skills to implement the Four Pillars Approach in managing pregnant women with anaemia. Finally, during the period of implementation of the Four Pillars Approach in Public Health Centres (data collection); the research team will periodically monitor and supervise this.

### Data analysis and model building

Double entry of data will be performed in Epidata [27]. Data will be transferred to SPSS and validated in SPSS (version 20) [28] where all statistical analysis will be conducted. Descriptive statistics will be used to describe the study population.

As the pregnant women are clustered within Health Care Centres, the intracluster dependence of the outcomes of the intervention will be assessed by calculating Intracluster Correlation Coefficients (ICC’s) [29].

A non zero ICC will lead to using random intercept generalized mixed models for analyzing the results of the Four Pillars Approach: a multilevel logistic model for binary outcomes (skilled birth attendance in birthing process) and a multilevel linear regression analysis for continous outcomes (Hb level). Count data (frequency of antenatal care visit) will be analyzed by multilevel linear regression analysis as an approximately normal distribution will be expected.

When the ICC equals zero logistic regression and a general linear model will be used to model the outcomes of the study.

The model will include intervention yes/no as well as patient characteristics as possible confounders. The baseline T0 Hb level will be entered when modelling the Hb level at T1. The results will be expressed as difference at T1 for Hb level, odds ratio for skilled birth attendance and difference in number of antenatal care visits with 95% confidence intervals. Statistical significance is established at p-value = 0.05. Explained variance (R-squared) for the linear model and the multilevel logistic counterpart thereof [29] will be reported as model fit statistic.

Analyses will be performed according to the intention-to-treat principle.

ATLAS.ti will be used to support the analysis of the qualitative data from the semi structured interviews on nurse-midwives’ perception of their experiences in managing pregnant women with anaemia, as well as the barriers and facilitating factors of the implementation of the Four Pillars Approach.

### Power calculation

We need a total of 360 pregnant women with anaemia: (1) to detect a minimum difference of Hb = 0.5 g/dl between the intervention and control groups after assuming a standard deviation of 1.01, with α = 0.05, a power of 0.80, ICC of 0.10 and a dropout percentage of 20%; (2) to detect an increase of 20% points in skilled birth attendance in labour, with a baseline (control) percentage of 50% and an Intra Class Correlation Coefficient (ICC) of 0.10 with α = 0.05, power of 0.80 and a dropout of 20%; and (3) to detect a mean difference in antenatal attendances of one visit between the intervention and control groups with standard deviation 2.1, with α = 0.05 and power 0.80, ICC of 0.10 and a dropout of 20% [30]. The sample size of 360 pregnant women with anaemia will consists of 180 pregnant women from intervention and other 180 pregnant women from control groups. The period of recruitment will be limited to three months, and every Public Health Centre will recruit at least seven pregnant women with anaemia consecutively.

## Discussion

The high prevalence of anaemia in pregnancy in Yogyakarta Special Province requires adequate nursing-midwifery care to prevent the adverse effects of anaemia in the perinatal period. Learning from the experiences of other low income countries in handling similar conditions, and considering the nurse-midwives’ views, we developed an innovative approach, called the Four Pillars Approach to antenatal care.

Our primary objective is to evaluate the effectiveness of the Four Pillars Approach in managing pregnant women with anaemia, indicated by a difference in haemoglobin level in pregnancy, improving the frequency of antenatal care attendance and skilled birth attendance during labour. In addition, the secondary objective of this study is to explore the barriers and the facilitating factors of the implementation of the Four Pillars Approach and to evaluate the patient’s and nurse-midwives’ satisfaction with the Four Pillars Approach.

The Four Pillars Approach will be implemented by the trained nurse-midwives in the Public Health Centres in Yogyakarta Special Province. It is expected that this approach will be effective in managing anaemia in pregnancy in order to prevent perinatal morbidity and mortality. If the Four Pillars Approach is effective in improving the outcome of pregnant women with anaemia, this approach could be implemented nationwide and be taken into consideration to improve the outcome for other conditions in pregnancy, after further research.

### Ethical approval

The Ethical Committee of the Faculty of Medicine, Universitas Gadjah Mada – Yogyakarta, has assessed and given the ethical approval for this study.

## Competing interests

The authors declare that they have no competing interests.

## Authors’ contributions

WW is a PhD student and first author of this manuscript and wrote the first draft of the manuscript. SJ, HB, RS, JvD, and ALMLJ revised the manuscript critically. All authors read and approved the final manuscript.

## Authors’ information

WW is a maternity nurse and PhD student at the Department of Gender & Women’s Health, Primary and Community Care, Radboud University Nijmegen Medical Centre, The Netherlands. SJ is a midwife PhD researcher at Royal Dutch Organisation of Midwives (KNOV), Utrecht, The Netherlands. HB is a statistician at the Department of Gender & Women’s Health, Primary and Community Care, Radboud University Medical Centre Nijmegen, The Netherlands. RS is an Obstetric Consultant at the Sardjito Hospital, Medical Faculty Universitas Gadjah Mada Yogyakarta, Indonesia. JvD is an Obstetric Consultant at the Radboud University Medical Centre Nijmegen, The Netherlands. ALMLJ is a family physician and academic professor of Gender & Women’s Health at the Radboud University Medical Centre Nijmegen, The Netherlands.

## Pre-publication history

The pre-publication history for this paper can be accessed here:

http://www.biomedcentral.com/1471-2393/14/163/prepub
